# Gene Model Annotations for *Drosophila melanogaster*: The Rule-Benders

**DOI:** 10.1534/g3.115.018937

**Published:** 2015-06-24

**Authors:** Madeline A. Crosby, L. Sian Gramates, Gilberto dos Santos, Beverley B. Matthews, Susan E. St. Pierre, Pinglei Zhou, Andrew J. Schroeder, Kathleen Falls, David B. Emmert, Susan M. Russo, William M. Gelbart, the FlyBase Consortium

**Affiliations:** * Department of Molecular and Cellular Biology, Harvard University, Cambridge, Massachusetts 02138,; † Department of Genetics, University of Cambridge, Cambridge CB2 3EH, United Kingdom,; ‡ Department of Biology, Indiana University, Bloomington, Indiana 47405, and; § Department of Biology, University of New Mexico, Albuquerque, New Mexico 87131

**Keywords:** bicistronic, stop-codon suppression, multiphasic exon, shared promoter, non-AUG translation start

## Abstract

In the context of the FlyBase annotated gene models in *Drosophila melanogaster*, we describe the many exceptional cases we have curated from the literature or identified in the course of FlyBase analysis. These range from atypical but common examples such as dicistronic and polycistronic transcripts, noncanonical splices, *trans*-spliced transcripts, noncanonical translation starts, and stop-codon readthroughs, to single exceptional cases such as ribosomal frameshifting and HAC1-type intron processing. In FlyBase, exceptional genes and transcripts are flagged with Sequence Ontology terms and/or standardized comments. Because some of the rule-benders create problems for handlers of high-throughput data, we discuss plans for flagging these cases in bulk data downloads.

The *D. melanogaster* genomic sequence assembly is of exceptionally high quality (Celniker *et al.* 2002; Hoskins *et al.* 2007, 2015) and is one of the few for which gene models have been manually annotated and assessed for all protein-coding and lncRNA genes (Matthews *et al.* 2015, which is the companion to this article). This has allowed FlyBase (dos Santos *et al.* 2015) to more easily identify and handle the rule-benders: gene models that incorporate exceptional or atypical transcription, splicing, or translation events. We summarize our current catalog of such exceptional gene models and events, including polycistronic transcripts, noncanonical splices, *trans*-spliced transcripts, noncanonical translation starts, stop-codon readthroughs, multiphasic coding exons, and ribosomal frameshifting. It is hoped that this extensively vetted compilation will support opportunities for further investigations into some of these rule-bending phenomena, including their biological impact, mechanistic bases, regulation, evolutionary development, and phylogenetic distribution.

## Materials and Methods

The FlyBase gene model set consists entirely of manually annotated transcripts, with the exception of some classes of small noncoding RNAs. Gene model annotation guidelines and datasets informing the gene annotation process are described in Matthews *et al.* (2015). All data and gene models are available at FlyBase (http://flybase.org).

## Results and Discussion

### Exceptional cases are flagged at the gene and transcript levels

Atypical gene models, those that do not follow the canonical rules, can create confusion among biologists accustomed to better-behaved genes and can cause unexpected errors in bulk data assessments. Some categories, notably *trans*-spliced transcripts, are frequently assumed to be errors. Overlapping genes complicate the design of sequence-based reagents (Hu *et al.* 2013) and global assessments of levels of gene expression. To flag these exceptions for users, FlyBase identifies known cases of rule-bending gene models at several levels. Appropriate Sequence Ontology (SO) terms (Eilbeck *et al.* 2005) are associated with the gene records (Table 1). These may be found in the gene reports in the “Sequence Ontology: Class of Gene” subsection under the “Gene Model and Products” section. This is a controlled field with links from the terms to the FlyBase Vocabularies tool. A comment in the “Comments on Gene Models” field includes the relevant SO term and often additional information regarding the nature and attribution of the exception. Standardized comments have been added to individual rule-bending transcripts when appropriate (Supporting Information, Table S1); FlyBase is in the process of adding similar comments to the transcript headers in our bulk data files (Table 2). Flags of the type “translation exception” are particularly important because they break the rules by which a predicted protein is derived from an annotated transcript. Exceptional cases are also flagged in GenBank RefSeq transcript and protein entries (Table S2). Each of the following sections concludes with a description of the SO, transcript and GenBank flags used for that type of exceptional gene model.

**Table 1:** Gene-associated Sequence Ontology terms

SO Term	SO ID Number
gene_with_dicistronic_mRNA	SO:0000722
gene_with_polycistronic_transcript	SO:0000690
gene_with_trans_spliced_transcript	SO:0000459
gene_with_unconventional_translation_start_codon	SO:0001739
gene_with_translation_start_codon_CUG	SO:0001740
gene_with_stop_codon_redefined_as_selenocysteine	SO:0000710
gene_with_stop_codon_read_through	SO:0000697
gene_with_transcript_with_translational_frameshift	SO:0000712

**Table 2:** Proposed transcript-associated flags to be included in FASTA files

Proposed Flag	Type
dicistronic_mRNA	Transcript exception
polycistronic_transcript	Transcript exception
non_canonical_splice_site	Transcript exception
endonuclease_spliced_intron	Transcript exception
trans_spliced_transcript	Transcript exception
non-canonical_start_codon	Translation exception
stop_codon_redefined_as_selenocysteine	Translation exception
stop_codon_read_through	Translation exception
transcript_with_translational_frameshift	Translation exception
mitochondrial_genetic_code	Translation exception
mitochondrial_incomplete_stop_codon	Translation exception
start_codon_not_determined	Translation exception
mutation in strain	Sequence alteration
genomic sequence error or gap	Sequence alteration

### Exceptional transcript structure (1): polycistronic (primarily dicistronic) transcripts are not uncommon

One of the surprising results of the first manual gene model annotation sweep of *D. melanogaster* performed by FlyBase (Misra *et al.* 2002) was the number of new dicistronic transcripts. At that time nine dicistronic loci had been previously described (Pauli *et al.* 1988; Schulz *et al.* 1990; Andrews *et al.* 1996; Brogna and Ashburner 1997; Ibnsouda *et al.* 1998; Niimi *et al.* 1999; Gray and Nicholls 2000; Liu *et al.* 2000; Walker *et al.* 2000). Misra *et al.* (2002) expanded the number to 31 dicistronic gene pairs that were confidently identified and another 17 that were tentatively identified. Dicistronic loci are no longer a surprise: there are 159 dicistronic gene pairs in annotation release 6.04. In addition, there are nine sets with transcripts encoding more than two genes (seven tricistronic and two tetracistronic) and one complex case involving overlapping dicistronics (of *seq*, *Kdm4B*, and *CG17724*). In total, 350 genes are annotated as sharing one or more transcripts with a neighboring protein-coding gene. Thus, 2.5% of protein-coding gene models include at least one polycistronic transcript. Note that FlyBase annotates each member of a dicistronic or polycistronic locus as a separate gene; this allows unambiguous association of gene-specific information, such as protein domains, molecular function, and mutant phenotypes. A listing of all polycistronic genes with additional information is found in File S1. [The *mod(mdg4)* polycistronic *trans*-splicing precursors are excluded from these totals and from this discussion; see description of *trans*-splicing below.]

In 2002, annotation of dicistronic loci was dependent on isolation of cDNA clones that spanned both genes. Now, RNA-Seq coverage data (Graveley *et al.* 2011; Brown *et al.* 2014) and whole genome assessments for transcription start sites (Hoskins *et al.* 2011; Batut *et al.* 2013) provide independent evidence of transcript extent and structure. Originally, some polycistronic genes were missed because it was difficult to determine which of many small open reading frames (ORFs) might correspond to functional protein-coding genes. Since then, the availability of genomic sequence information for multiple *Drosophila* species has allowed identification of some conserved ORFs as small as 20–30 amino acids (Lin *et al.* 2007, 2011; Hayden and Bosco 2008).

**Figure 1:**
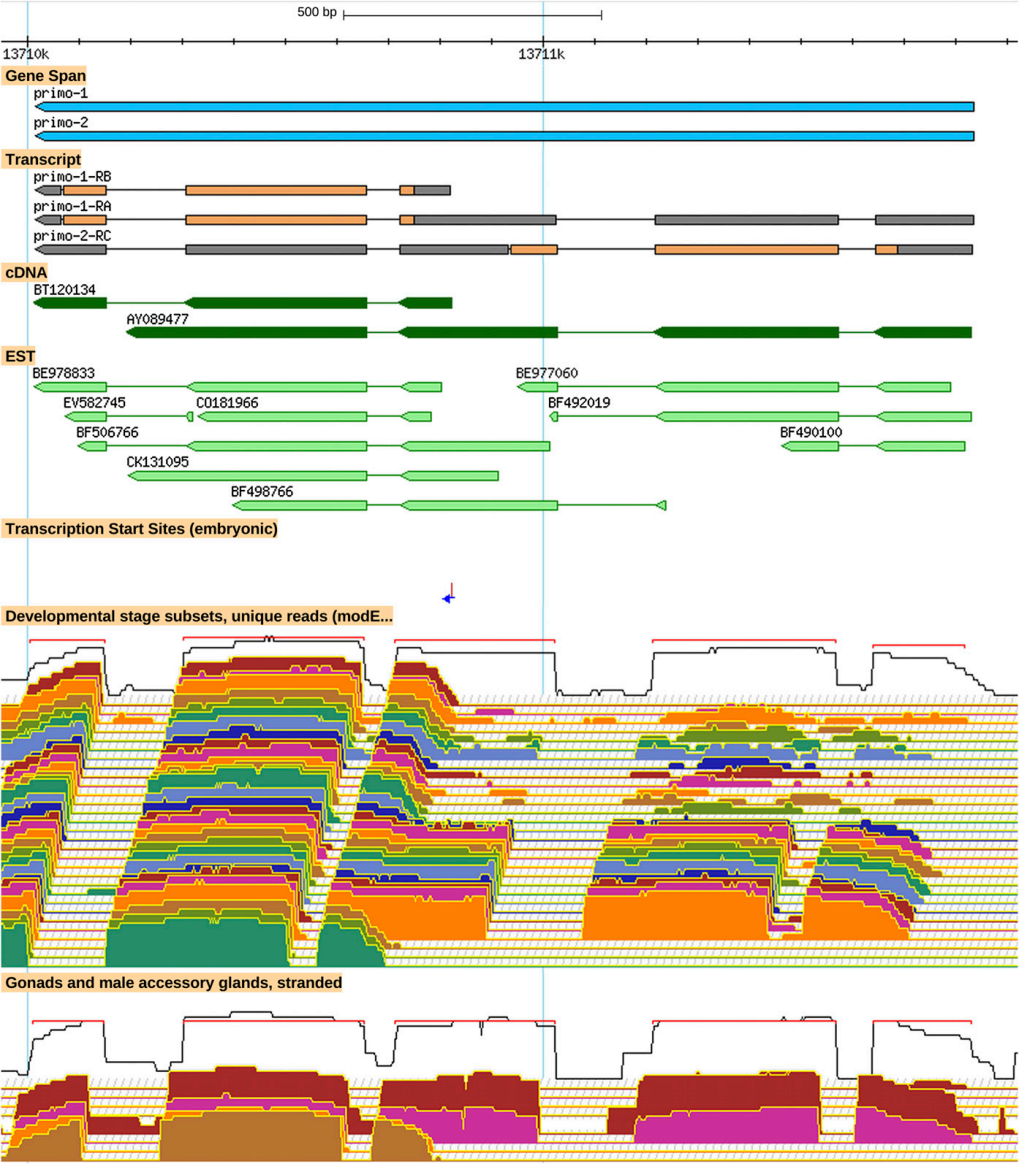
A dicistronic transcript isoform for *primo-1* and *primo-2* is produced from a stage- and tissue-specific promoter. A GBrowse view showing (top to bottom): the gene extents and the gene models; cDNAs and ESTs; transcription start site(s); unstranded RNA-Seq coverage data corresponding to a developmental series (early embryos, top, to adults, bottom); and stranded RNA-Seq coverage data (plus strand top, minus strand bottom) corresponding to testis (red), male accessory gland (magenta), ovary from virgin females (orange), and ovaries from mated females (tan). More information on data presented in GBrowse may be found at http://flybase.org/wiki/FlyBase:GBrowse_Tracks#General.

Polycistronic loci often encode alternative transcripts that are monocistronic. Of the 159 currently annotated dicistronic loci, there are only 45 for which polypeptides from both genes of the pair appear to be produced exclusively from dicistronic transcripts. There are also 45 loci for the opposite case, with support for alternative monocistronic transcripts for both genes. In some of these cases, the dicistronic transcript appears to be produced at a relatively low frequency or only in specific circumstances. For the remaining 69 loci, one gene has both monocistronic and dicistronic transcripts and the other is encoded only by dicistronic transcripts (example in Figure 1). The nine loci that include tricistronic and tetracistronic transcripts may encode a mix of polycistronic types and several also include an alternative monocistronic transcript (example in Figure S1). Although we cannot be certain that we have identified all alternative monocistronic transcripts, currently 181 gene models include only polycistronic transcript isoforms. Even if this is a significant overestimate, it is likely that more than 1% of protein-coding genes in *D. melanogaster* are encoded exclusively by polycistronic transcripts.

Genes that encode small polypeptides are over-represented among polycistronic genes. There are 10 genes for which all annotated polypeptides are less than 25 amino acids; all are polycistronic, although one is also annotated with an alternative monocistronic transcript. Of the 65 genes for which all annotated polypeptides are between 25 and 45 amino acids, 15 (23%) are polycistronic. A number of genes in this category correspond to small conserved ORFs found in the 5′ or 3′ untranslated regions (UTRs) of longer coding genes (Hayden and Bosco 2008); these are defined as separate genes in FlyBase. There are undoubtedly additional translated ORFs less than 50 amino acids that will be identified by new techniques, such as ribosomal profiling combined with proteomic validation (see Gawron *et al.* 2014 for discussion of emerging approaches), so the list of polycistronic genes encoding small polypeptides is likely to grow.

Polycistronic loci are not a homogenous group (File S1). The distance between the upstream stop codon and the downstream start codon ranges from negative (overlapping in different ORFs) to over a kilobase. For loci that produce only polycistronic transcripts (no alternative monocistronics), the range between upstream stop and downstream start is −34 nt to 319 nt. Alternative monocistronic transcripts for the 3′ coding region are usually transcribed from an alternative downstream (or external) promoter; monocistronic transcripts for the 5′ coding region usually terminate at an upstream polyadenylation site. However, a number of alternative monocistronic transcripts are the result of alternative splices that disrupt one of the two coding regions. Approximately 18% of polycistronic loci include similar genes; many of these may have been created by tandem duplication. Among the other 82%, a few are known to be functionally related (Liu *et al.* 2000; Phillips *et al.* 2000), including the highly conserved example of the large and small subunits of Molybdopterin synthase 2, *Mocs2* and *CG42503* (Hayden and Bosco 2008).

Ben-Shahar *et al.* (2007) present preliminary evidence that the *CheB42a* and *ppk25* genes are transcribed as a dicistronic pre-mRNA; they postulate that this pre-mRNA undergoes a unique cleavage event to produce two monocistronic mRNAs. This proposed mechanism differs from the polycistronic loci we define, for which polycistronic processed mRNAs are supported, and for which each cistron’s product is postulated to be translated from a single mRNA isoform. The gene models we describe as polycistronic necessitate internal initiation of translation for the downstream gene by use of an internal ribosome entry site (IRES) (reviewed in Hellen and Sarnow 2001), resumption of ribosomal scanning (Wall *et al.* 2005), or some other mechanism. Use of an IRES may be common, because although some dicistronic pairs appear to meet the requirements of a ribosomal scanning mechanism, most do not [see the discussion in Misra *et al.* (2002) and additional information tabulated in File S1].

#### FlyBase and GenBank flags:

Genes with dicistronic or polycistronic transcripts are identified by specific SO terms (Table 1). At the transcript level, comments are included in FlyBase transcript reports (Table S1), and a “dicistronic gene” exception is included in the GenBank RefSeq transcript entry (Table S2). The proposed bulk data flag is of the “transcript exception” type (Table 2).

### Exceptional transcript structure (2): adjacent genes may share exons, noncoding and coding

There are many cases in which genes on the same strand overlap each other. The most extreme cases are the polycistronic gene models, as described above. Two categories we have not viewed as sufficiently unruly that they merit special treatment or comments: a gene that is nested in the intron of a gene on the same strand or a gene with a 3′ UTR that overlaps the 5′ UTR of the downstream gene. Intermediate cases in which two genes share an exon, usually including a shared splice site, are described below. See File S2 for a complete listing of the genes discussed in this section.

The first example in the intermediate category is that of shared exons, but not shared coding DNA sequence (162 gene models flagged). Most common are cases in which two genes share a promoter and 5′ noncoding exons (130 genes); this includes 13 pairs for which one gene is coding and the other is noncoding. In some cases, the shared promoter is used by all transcripts of both genes, for example, *Ip259* and *RpS27A*. In other cases, for example, *CG2911* and *Spec2*, one or both gene models include transcripts derived from unshared promoters. Genes may also share 3′ UTRs (14 genes); an extreme example is the set comprising *inaF-A*, *inaF-B*, *inaF-C*, and *inaF-D* (Cheng and Nash 2007). There are eight sets (18 genes) described as complex or atypical cases, usually in which both 5′ and 3′ UTRs overlap.

The second example in the intermediate category consists of genes that share exons including a short extent of the coding sequence (in the same open reading frame), usually at the amino terminus. Historically, FlyBase categorized any transcripts that shared any amount of coding sequence as belonging to one gene. This policy has been changed for a small number of cases in which the genes in question are clearly functionally and evolutionarily distinct. Of the cases currently annotated (10 pairs and one triplet), most of the genes share a promoter, the first exon, and a translation start for at least one pair of transcript isoforms; some of these genes also have alternative transcript isoforms with different promoters that do not share coding sequences with the second gene.

A well-studied case of such coding sequence (CDS) overlap is that of *Su(var)3-9* and *eIF-2γ* (Krauss *et al.* 2006), which may have resulted from the transposition of *Su(var)3-9* into an intron of *eIF-2γ*. The two genes share a promoter, translation start, and 80 N-terminal residues that are similar to the N-terminus of eIF-2γ-like proteins in other species. The two genes, however, encode polypeptides with very different functions: histone methylation and translation initiation. This gene fusion encodes the only *D. melanogaster* orthologs for two different highly conserved eukaryotic genes. There is no transcript isoform for either gene that includes the characteristic domains of both *Su(var)3-9* and *eIF-2γ*. The gene fusion appears to be insect-specific, with instances of re-fission in aphids (Krauss *et al.* 2006).

#### FlyBase flags:

Several comments are used to flag FlyBase gene models with shared noncoding exons: “Shares 5′ UTR,” “Shares 3′ UTR,” “Shares 5′ exon(s),” and “Complex/atypical overlap,” followed by additional explanatory information. Genes that contain overlapping coding extents are flagged with gene model comments that begin “Genes with CDS overlap,” followed by additional specific information. The affected transcripts are not flagged; an exceptional translation flag is not necessary.

### Exceptional transcript structure (3): overlapping genes or alternative transcripts may share multiphasic or bidirectional regions of coding sequence

Coding regions for which more than one overlapping ORF on the same strand appears to be used are described as “multiphasic.” There are 269 gene models flagged with a comment indicating that the current annotation includes transcripts that share a multiphasic region; a complete listing is available in File S3. These annotations are based on data supporting different transcript isoforms; in most cases, there are no data addressing whether both protein isoforms are biologically relevant. The majority of cases correspond to transcripts that include a variably spliced intron that results in a frameshift in the next exon, a short multiphasic extent, and an alternative stop codon. If the multiphasic extent is less than 40 nucleotides (N = 133), we flag the gene model with the comment “Alternative translation stop created by use of multiphasic reading frames within coding region.” Because the nucleotide extent includes the stop codon, this corresponds to less than 13 amino acids. In some instances, the frameshift produces a significantly truncated protein, for example, *Ucp4B* and *Start1*; in others, the resulting change in carboxy sequence appears to be minor, for example, *CG15278*. Transcripts with a trunctated CDS are flagged with an additional comment that concludes “results in premature stop codon and/or downstream start; may or may not produce functional polypeptide” (see Matthews *et al.* 2015). The multiphasic extent may be longer than 40 nucleotides (N = 118) or involve the initial coding exon (N = 18); and examples include *NetA* and *CG31948*. These are flagged with the comments “Multiphase exon postulated: exon reading frame differs in alternative transcripts” or “Multiphase exon postulated: reading frame of first coding exon differs in alternative transcripts.” For 83 genes, the annotated multiphasic overlap exceeds 63 nucleotides (>20 amino acids); the longest is the 704-nucleotide multiphasic overlap found for transcripts of the *moody* gene (Bainton *et al.* 2005). For several genes, production and function of both alternative protein isoforms derived from a multiphasic exon have been demonstrated (including *Ada2b*, Pankotai *et al.* 2013; *moody*, Bainton *et al.* 2005; and *Xbp1*, a unique case described below).

Multiphasic regions may involve two genes. There are 36 genes (18 pairs) flagged with the comment “Multiphase exon postulated: this gene shares a region of coding sequence with an overlapping gene, but different reading frames are utilized in the overlapping coding region.” Of these, 26 are encoded on polycistronic transcripts. Most overlap by less than 40 nucleotides; however, for five pairs the multiphasic overlap exceeds 63 nucleotides. The most dramatic example is the *att-ORFA* and *att-ORFB* pair, which overlaps for 553 nucleotides across two exons and for which there is evidence that both protein products are produced (using an *in vitro* translation assay) (Madigan *et al.* 1996). Currently, there is a single pair of genes annotated with overlapping coding extents on opposite strands: *CG34148* and *P5CDh2*. We describe such a shared region as “bidirectional” and flag these genes with the comment “Bidirectional region of coding sequence postulated: a portion of the CDS of this gene overlaps a portion of the CDS of a gene on opposite strand.” Criteria for annotating bidirectional genes are discussed in Matthews *et al.* (2015).

#### FlyBase flags:

Genes with multiphasic exons or bidirectional regions are flagged with the comments described above. The affected transcripts are not flagged; an exceptional translation flag is not necessary.

### Exceptional splicing (1): noncanonical splices are rare

For 99% of the ∼60,000 annotated introns, the primary canonical splice donor-acceptor pair GT-AG is used; for most of the remaining 1%, the secondary canonical splice donor-acceptor pair GC-AG is used (563 GC-AG out of 60,223 total in release 6.03). Thus, the number of introns for which other splice donor-acceptor pairs are used is very low: only 79 are annotated in current gene models (a complete listing is available in File S4). Considering the high levels of cDNA and RNA-Seq junction data that have been incorporated into the current gene model annotations, it is likely that most typically used noncanonical splices have been identified. Introns supported only by very low-frequency data are not usually included in FlyBase gene models (Matthews *et al.* 2015); there are some additional noncanonical splices in this category, primarily representing potential alternatively spliced introns within 5′ UTRs. Introns processed by the U12 spliceosome have been well characterized (Schneider *et al.* 2004; Alioto 2007; Lin *et al.* 2010); remarkably, many are conserved between flies and humans (Lin *et al.* 2010). Most of the AT-AC introns in the current annotation set are of the U12 type (Table 3) and had been previously identified.

**Table 3:** Introns with noncanonical splice sites and/or U12-type 5′ consensus sequence

Splice Donor-Acceptor Pair	Number in Release 6.04	Number with RNA-Seq Junction Support	Number with Similar Alternative Splice	Within Coding	Within 5′ UTR	Within lncRNA
AT-AC (U12)	9	9	1	9	0	0
AT-AC (U2)	4	4	2	4	0	0
GT-TG	23	19	22	13	9	1
GT-GG	6	5	5	3	2	1
GT-CG	8	8	8	6	2	0
GT-AT	14	11	14	11	3	0
GT-AA	3	3	3	2	1	0
GA-AG	12	12	5	8	3	1
GG-AG	0	—	—	—	—	—
GT-AC	0	—	—	—	—	—
**Total**	**79**	**71**	**60**	**56**	**20**	**3**
GT-AG (U12)	10	9		9	1	0
GC-AG (U12)	1	1		1	0	0

**Figure 2:**
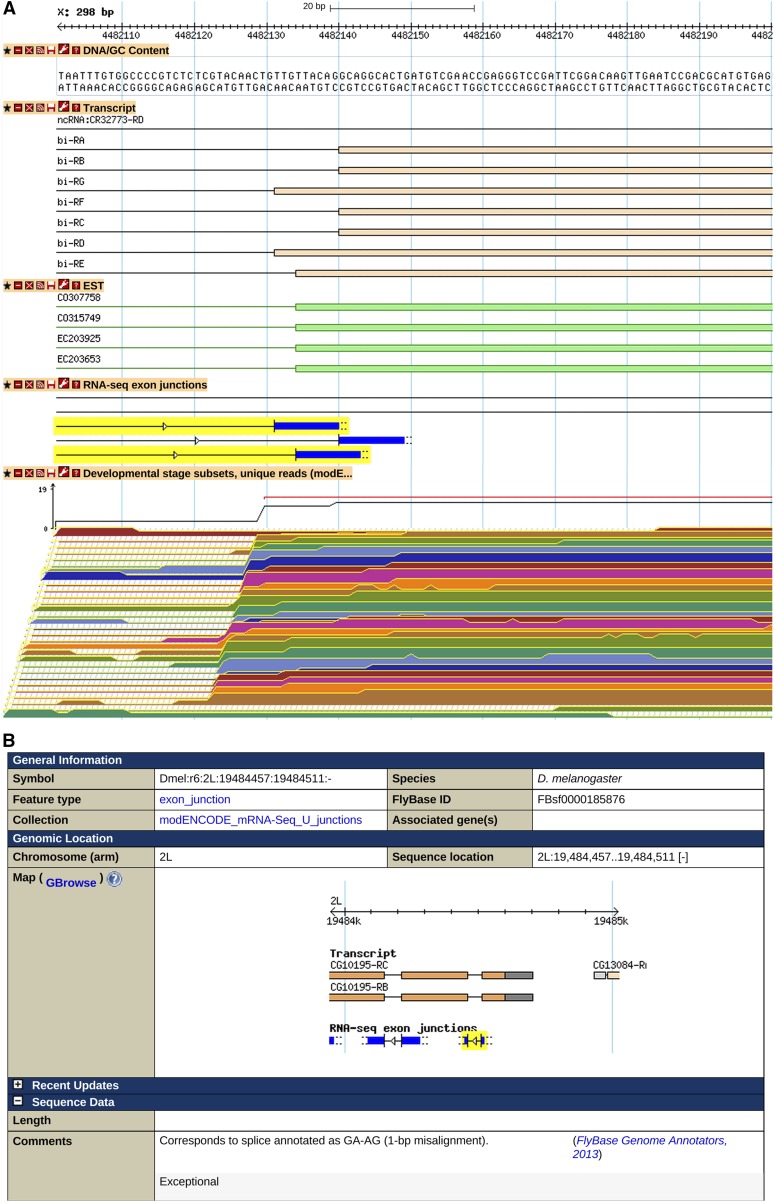
Noncanonical splices supported by RNA-Seq junction data. (A) Of three alternative splice acceptors for intron 6 of the *bifid (bi)* gene, two are noncanonical TGs, including the splice acceptor used at the highest frequency (first highlighted junction). A GBrowse view showing (top to bottom): nucleotide sequence; region of the gene model showing one intron/exon boundary; EST data; RNA-Seq junction data; and unstranded RNA-Seq coverage data corresponding to a developmental series (early embryos, top, to adults, bottom). More information on data presented in GBrowse may be found at http://flybase.org/wiki/FlyBase:GBrowse_Tracks#General. (B) Report for an RNA-Seq junction that corresponds to a noncanonical splice but is aligned to incorrect noncanonical sites, one of several cases that were slightly misaligned.

With the exception of AT-AC (and the mechanistically different HAC1-type intron splice sites, see below), the noncanonical splice sites currently annotated in *D. melanogaster* differ from GT-AG by only one base, with the G of the donor site being invariant. Of the eight possible pairs (excluding GT-AG and GC-AG) that fit these criteria, six are observed (Table 3). The distribution among genes is nonrandom: 10 genes are annotated with more than one intron defined by a noncanonical splice pair, despite the very low frequency of such introns (File S4). For the majority of cases there is a similar alternative canonical splice (Table 3); this has also been observed in humans (Szafranski *et al.* 2007; Parada *et al.* 2014). Although in some cases this may be a mechanism to increase protein variation (example in Figure 2A), similar alternative splices are also observed for splices that occur in 5′ UTRs. The key exceptions to this pattern of similar alternative splices are U12 spliceosome introns and GA-AG introns.

Noncanonical splices frequently foil gene model prediction programs and complicate cDNA alignments and algorithms for deriving RNA-Seq junction data. Currently, 71 of the 79 annotated noncanonical splices have a supporting RNA-Seq junction (Table 3). For most (57 of 71), the alignment is impressively accurate; however, a number of the junctions are slightly misaligned and thus do not appear to map precisely to the annotated intron. A particular problem for unstranded RNA-Seq data are GT-AT splices, which are usually called as AT-AC splices on the opposite strand (eight of 10 cases). For exceptional junctions that correspond to annotated introns, the noncanonical acceptor/donor and any alignment inconsistencies are identified in explanatory comments in the FlyBase RNA-Seq junction reports (Figure 2B).

A more significant problem for identification of noncanonical splices by RNA-Seq junction analysis is false positives; including AT-AC calls, there are more than 500 exceptional junctions from the combined Baylor (Daines *et al.* 2011) and modENCODE datasets (Graveley *et al.* 2011). Some of these can be culled by eliminating low-frequency junctions and those with weak confidence scores; however, exceptional junctions within the middle ranges of frequency or confidence scores need to be manually assessed. Common problems (shared with nonexceptional junctions) include spurious calls within coding sequences comprising repeated motifs (for example, *CG10953* and *Eig71Ee*), mismatch of acceptor and donor calls across related tandem genes (for example, the Jon99C cluster and the trypsin cluster at 47F), atypical calls within very highly expressed genes (for example, *RpS13* and *Cyt-b5-r*), and RNA-Seq data mapping to unmasked repeat elements. A number of exceptional junction calls are one nucleotide off from a well-supported canonical splice; these are assumed to be artifactual.

Currently, there are approximately 40 transcripts in FlyBase that are annotated with noncanonical splices as a matter of convenience; most of these splices are unlikely to occur *in vivo* and are not included in the description of supported noncanonical splices above. They are used to allow representation of an intact gene model when the gene extent is interrupted by a transposable element insertion or a genomic sequence gap or is within a heterochromatic region that may vary from strain to strain. These cases are flagged with specific explanatory comments (Table S2).

#### FlyBase and GenBank flags:

Genes with transcripts annotated with noncanonical splices are not flagged; there is no appropriate gene-level SO term. Comments at the transcript level are included in FlyBase transcript reports (Table S1) and as a “nonconsensus splice site” exception in the GenBank RefSeq transcript and protein entries (Table S2). The proposed bulk data flag is of the “transcript exception” type (Table 2).

### Exceptional splicing (2): a single HAC1-type intron splice is highly conserved

Several rule-defying exceptional gene models are, in fact, examples of uncommon, yet very highly conserved, phenomena. One notable example is that of *Xbp1* (Ryoo *et al.* 2007), a gene with an alternatively spliced isoform that has a very short intron (23 nt) flanked by noncanonical splice junctions (CA-TG). This alternative isoform is translated into a protein with the carboxy terminus in a different reading frame with respect to the unspliced isoform. Although this looks like an especially egregious annotation error, it is actually an example of Ire1-mediated unconventional splicing, a process conserved from yeast to mammals as part of the unfolded protein response (UPR). In this response to ER degradation stress, the protein Ire1 acts as a sensor and mediates a signaling cascade; it also acts as an endonuclease with a single target: the HAC1 ortholog *Xbp1*. The unconventionally spliced *Xbp1* transcript is translated into a bzip transcription factor that upregulates genes responsive to ER stress. Like most organisms, *D. melanogaster* has exactly one example of the HAC1-type intron splice mechanism (Sidrauski and Walter 1997; Plongthongkum *et al.* 2007; Hooks and Griffiths-Jones 2011).

#### FlyBase and GenBank flags:

The *Xbp1* gene report includes an explanatory comment in the gene model comment section. The report for the transcript that results from Ire1-mediated splicing includes a similar explanatory comment (Table S1) plus the flag “endonuclease_spliced_intron” (Table 2). A “nonconsensus splice site” exception appears in the GenBank RefSeq transcript and protein entries (Table S2). The proposed bulk data flag is of the “transcript exception” type (Table 2).

### Exceptional splicing (3): *trans*-splicing is well supported for two genes

At least two genes in *D. melanogaster* undergo *trans*-splicing, a process by which a mature mRNA is created by a bimolecular splice between two independently transcribed pre-mRNAs. In both known cases, the gene encodes multiple DNA-binding proteins with a common amino BTB/POZ domain and variable carboxy zinc-finger domains. The initial and more dramatic example is *mod(mdg4)* (Labrador *et al.* 2001; Dorn *et al.* 2001), which encodes more than 30 protein isoforms, at least 18 of which appear to be *trans*-spliced. The second example is *lola*, a gene that encodes at least 20 protein isoforms, one of which shows evidence of being *trans*-spliced (Horiuchi *et al.* 2003). In recent work, Gao *et al.* (2015) have begun to elucidate the molecular mechanisms of *trans*-splicing in the *mod(mdg4)* and *lola* systems.

These two examples of *trans*-splicing are in the category of intragenic *trans*-splicing, in contrast to the better-characterized category of spliced leader (SL) *trans*-splicing events originally observed in trypanosomes and nematodes (reviewed in Lasda and Blumenthal 2011) and in contrast to intergenic *trans*-splicing, which generates chimeric mRNAs. Both intragenic and intergenic *trans*-splicing have been observed in mammalian cells, but these events appear to be rare and in some cases are associated with neoplastic cells (reviewed in Horiuchi and Aigaki 2006).

In the case of *mod(mdg4)*, five clusters of polycistronic transcripts encoding multiple 3′ alternative termini are supported by cDNA/EST and transcriptional start site data (Nechaev *et al.* 2010; Batut *et al.* 2013). These have been annotated as separate genes by FlyBase, with symbols such as *pre-mod(mdg4)-T* for the precursor of *mod(mdg4)-RT*. Two of these clusters are transcribed from the strand opposite to the rest of the *mod(mdg4)* exons, and are what precipitated the discovery of *trans*-splicing in *D. melanogaster*. It appears likely that this locus has undergone multiple small inversions since the *Drosophilidae* diverged from other dipterans: the genomic pattern of *trans*-encoded exons is observed in other *Drosophila* species (Gabler *et al.* 2005), but in mosquitoes it appears that all the 3′ alternative exons are located on the same strand (http://vectorbase.org, AGAP003439) (Krauss and Dorn 2004; Megy *et al.* 2012).

For *lola* there is clear support for at least one *trans*-spliced precursor, including EST and transcription start site data. It appears to be monocistronic and corresponds to the 3′-most alternative exon; it has been annotated as a separate gene in FlyBase, *pre-lola-G*.

Additional candidate genes subject to *trans*-splicing have been suggested but await definitive evidence (McManus *et al.* 2010). For *broad*, another gene encoding multiple isoforms with a constant BTB/POZ domain and variable zinc-finger domains, there are data supporting a transcription start site 5′ of the last two variable exons (Batut *et al.* 2013), suggesting that this gene may undergo *trans*-splicing. A number of complex gene model annotations include short 3′ isoforms with supported alternative downstream transcription starts; in some cases, there are also 5′ isoforms that do not overlap the 3′ isoforms (for example, *vir-1*, *CG43427*, *dlg1*). It is interesting to speculate that some of these loci may also be subject to *trans*-splicing.

#### FlyBase and GenBank flags:

Genes with *trans*-spliced transcripts, including the 3′ *trans*-splicing precursors, are identified by a SO term (Table 1). For the nine *mod(mdg4)* spliced transcripts derived from exons on opposite strands, a comment is included in FlyBase transcript reports (Table S1) and a “trans_splicing” comment appears in the GenBank RefSeq transcript and protein entries (Table S2). The proposed bulk data flag is of the “transcript exception” type (Table 2).

### Exceptional transcript modification: A-to-I RNA editing is noted at the genome level only

Post-transcriptional modifications of pre-mRNAs that result in changes to the nucleotide sequence are described as RNA editing. A-to-I RNA editing results from modification of an adenosine to inosine, which subsequently acts as would a guanine in terms of translation, splicing, and in the formation of secondary structures (reviewed in Mallela and Nishikura 2012). In *D. melanogaster*, 2005 putative A-to-I RNA editing sites have been identified and mapped on the genome, overlapping the transcripts of 1307 genes (Graveley *et al.* 2011; Rodriguez *et al.* 2012). A more recent study (St. Laurent *et al.* 2013) has not yet been incorporated into FlyBase. Most genes subject to A-to-I editing have at least three associated editing sites, and two genes have more than 20 overlapping editing sites (*para* is associated with 23, *NaCP60E* with 21). Because editing is developmentally regulated, and because the efficiency of each editing site varies, different combinations of editing are also possible for a given transcript, further adding to the vast number of possible permutations.

FlyBase does not attempt to represent alternative transcript or polypeptide sequences that may result from RNA editing. Identified A-to-I editing sites are annotated on the genome and are viewable in relation to gene model annotations on GBrowse. A Sequence Feature Report for each editing site details the observed editing frequency at various developmental stages, as well as the potential impact of a given editing site on the coding sequence of the related transcript (see, for example, A-I_edit_000244, FBsf0000383608). Each of these identified genomic sites is associated with the SO term “modified_RNA_base_feature” (SO:0000250). The SO term “gene_with_edited_transcript” (SO:0000548) is used to flag genes subject to A-to-I RNA editing.

### Exceptional translation (1): noncanonical translation starts have been difficult to identify

**Figure 3:**
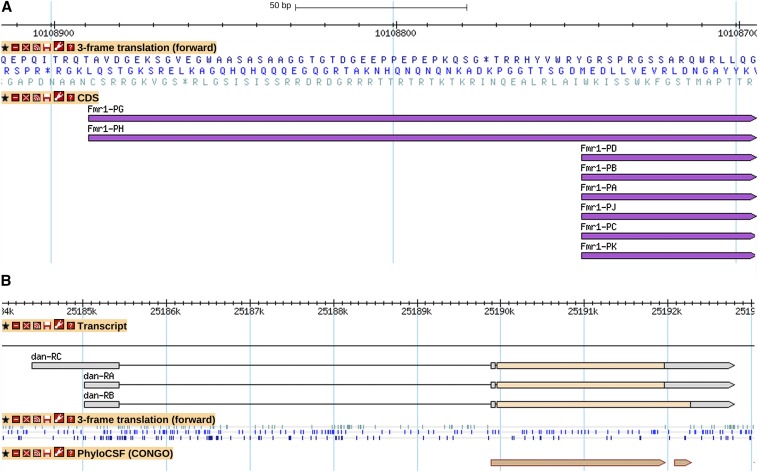
Noncanonical terminal extensions of the CDS. (A) CUG start codon in *Fmr1* results in a 48-aa N-terminal extension; a GBrowse view showing amino acid sequence and amino ends of annotated polypeptides. Use of this alternative start codon has been confirmed by Western blot, mutagenesis of reported constructs, and rescue constructs (Beerman and Jongens 2011). (B) For the *dan* gene model, a stop-codon readthrough annotated for *dan-RB* is supported by PhyloCSF analysis (conservation of protein signatures). A GBrowse view showing (top to bottom): the gene model; stop codons on the plus strand in each of the three open reading frames; and regions of protein conservation among the *Drosophila* species (tan extents at the bottom). More information on data presented in GBrowse may be found at http://flybase.org/wiki/FlyBase:GBrowse_Tracks#General.

Thus far, no systematic or definitive study of non-AUG translation initiation in *Drosophila* has been performed. Individual cases have been discovered more or less by chance; some of the more thoroughly characterized include *ChAT* (Sugihara *et al.* 1990), *ewg* (de Simone and White 1993), *Eip74EF* (Boyd and Thummel 1993), *Akt1* (Andjelkovic *et al.* 1995), and *Fmr1* (Figure 3A) (Beerman and Jongens 2011). The majority of the currently annotated cases, including all based on FlyBase analysis (Table S3), are postulated due to the lack of an appropriately placed AUG codon; most are supported by assessment of conservation among *Drosophila* species or a favorable sequence context for translation initiation, but no additional experimental data.

Recently, several systematic studies have allowed a more comprehensive characterization of noncanonical translation initiation in human and mouse. Searching for evolutionary signatures of protein-coding sequences within predicted 5′ UTRs, Ivanov *et al.* (2011) found 42 new and confirmed 17 previously reported non-AUG starts in humans. All are near-cognates (differing by one base) of AUG; CUG is by far the most common (42%). It may be a requirement that the second base is a pyrimidine, because AAG and AGG starts were not found. Ribosome profiling in mouse embryonic stem cells (Ingolia *et al.* 2011) indicates that for many transcripts multiple translation starts may be used, primarily additional AUG sites, but also near-cognate codons. Again, CUG was found to be the most common noncanonical start codon. It appears that during translation a non-AUG start codon is still paired with the usual initiating tRNA, Met-tRNAi, and that the protein initiates with a methionine (Peabody 1989; Menschaert *et al.* 2013). Thus, in FlyBase, the predicted protein sequence from a non-AUG translation start initiates with a methionine, not the amino acid that is usually associated with that codon.

In FlyBase, there are currently 27 genes with transcripts annotated with a non-AUG translation start codon; 11 of these use a CUG start codon (Table S3). However, it seems likely that there are, in fact, many-fold more, especially in the category of alternative translation starts. There are a significant number of gene models for which an alternative splice or an alternative 5′ exon removes all in-frame AUG translation starts within the amino end of the putative protein. Most of these are currently annotated as short protein isoforms (using a downstream AUG), but may in fact be instances in which a non-AUG start is used. It is interesting that the amorphic *y*^*1*^ mutation is caused by a replacement of the AUG start codon with a CUG codon (Matthews *et al.* 2015). Despite the fact that CUG is the mostly widely used noncanonical translation start, it is unable to function as a start codon in this context.

#### FlyBase and GenBank flags:

Genes with transcripts annotated with noncanonical translation starts are flagged with SO terms (Table 1) in the gene model comment section. Comments at the transcript level are included in FlyBase transcript reports (Table S1); a “non-AUG translation initiation” comment and a translation exception flag appear the GenBank RefSeq transcript and protein entries (Table S2). The proposed bulk data flag is of the “translation exception” type (Table 2).

### Exceptional translation (2): few selenoproteins are found in flies

Selenocysteine stop-codon readthrough is another example of rule-bending on a translational level: a UGA codon is read not as a stop signal, but rather as a 21^st^ amino acid, selenocysteine (Sec; “U” in the single-letter code) (Castellano *et al.* 2001). Selenoprotein synthesis requires a specialized tRNA, Sec-tRNA^sec^, as well as the proteins involved in the synthesis of Sec-tRNA^sec^. The selenocysteine UGA codon is distinguished from a UGA stop codon by the presence of downstream stem-loop structure, the Sec insertion sequence (SECIS) in the 3′ UTR of the transcript, which is recognized by SECIS-binding protein (Martin-Romero *et al.* 2001). Selenocysteine is not only a means to allow a stop-codon readthrough; it also has a catalytic advantage over cysteine in the active site of oxidoreductases (Driscoll and Chavatte 2004).

*D. melanogaster* has three identified selenoprotein genes: *BthD*, *SelG*, and *Sps2*, which were identified by both *in silico* analysis of the *D. melanogaster* genome (Castellano *et al.* 2001) and by metabolic labeling with ^75^Se (Martin-Romero *et al.* 2001). *BthD* and *Sps2* have the Sec insertion quite early in the final protein; *SelG*, however, extends only two amino acids past the substituted stop codon. Selenoproteins are less well conserved than some of the more rare phenomena: flies have three, humans have 25, worms have only one, and yeast and higher plants lack them altogether. Many of the mammalian selenoproteins themselves are conserved, but the invertebrate nonselenoprotein orthologs have a cysteine in place of Sec (Driscoll and Chavatte 2004). In fact, at least two of the *D. melanogaster* selenoprotein genes have nonselenoprotein paralogs genomically nearby (Castellano *et al.* 2001). In contrast, prokaryotes and archaebacteria have a completely different complement of selenoproteins (Driscoll and Chavatte 2004).

#### FlyBase and GenBank flags:

Selenoprotein genes are identified by a specific SO term (Table 1). At the transcript level, comments are included in FlyBase transcript reports (Table S1) and a translation exception flag appears in the GenBank RefSeq transcript and protein entries (Table S2). The proposed bulk data flag is of the “translation exception” type (Table 2).

### Exceptional translation (3): stop-codon readthrough appears to be common in flies

Stop-codon readthrough is a well-documented regulatory mechanism in viruses (reviewed in Bertram *et al.* 2001) and has been investigated in *S. cerevisiae* (reviewed in von der Haar and Tuite 2007). Prior to 2007, a small number of specific cases had been identified in flies, including *kel* (Xue and Cooley 1993; Robinson and Cooley 1997), *oaf* (Bergstrom *et al.* 1995), *Syn* (Klagges *et al.* 1996) and *hdc* (Steneberg *et al.* 1998). In 2007, using a genome-wide comparative analysis designed to detect regions exhibiting evolutionary signatures specific to protein-coding regions (PhyloCSF) (Lin *et al.* 2007, 2011), a surprising number of such regions was found immediately beyond annotated stop codons. Because there are other possible explanations for this observation (alternative splicing, A-to-I RNA editing, or polycistronic transcripts, for example), a more thorough analysis was performed by Jungreis *et al.* (2011). Based primarily on these comparative evolutionary analyses, 328 genes are currently annotated in FlyBase with one or more transcripts subject to stop-codon readthrough (example in Figure 3B); a complete listing is available in File S5. In 22 cases a double readthrough is supported; there even appear to be two cases of triple readthrough (*vvl*, *Ets65A*). Two genes exhibit readthrough at two independent sites within alternative exons (*Oamb* and *CG34377*). The *Oamb* gene model is a particularly enthusiastic example of stop-codon readthrough: it has two independent sites, one of which is a double readthrough.

A number of predicted cases of stop-codon readthrough have been confirmed by mass spectrometry of wild-type proteins (seven cases) or using reporter constructs in which the readthrough extensions are epitope-tagged (Jungreis *et al.* 2011). Additionally, an independent ribosome-profiling assay using early embryos and S2 cells has confirmed 43 of the previously predicted readthrough cases (Dunn *et al.* 2013); it is unclear if unconfirmed cases occur at low levels or only at other developmental stages. Interestingly, this ribosome profiling study identified an additional 307 cases of translation readthrough not identified by Jungreis *et al.* (2011). These novel cases of translation readthrough exhibit lower conservation scores and lower readthrough rates than those predicted by conservation; the nucleotide character of these novel readthrough regions is intermediate between coding regions and 3′ UTRs. As such, it is unclear if these extensions represent truly functional isoforms of the proteins, or simply provide fodder for the evolution of novel C-terminal variants. For this reason, poorly conserved cases of readthrough are not currently annotated by FlyBase.

With very few exceptions, the annotated readthroughs result in a C-terminal extension of an annotated polypeptide sequence (as opposed to the addition of upstream coding sequences to an annotated polypeptide); note that the analysis of Jungreis *et al.* (2011) was biased to detect this type of event. This stop-codon readthough phenomenon appears to be distinct from the selenocysteine system and does not require a downstream SECIS motif (Jungreis *et al.* 2011). The annotated stop-codon readthroughs include cases for all three stop codons, with UGA being the most common. The length of the amino acid extension ranges from four amino acids to more than 1000 amino acids; most are within the range of 8–300 amino acids. Comparisons across 12 *Drosophila* species for individual genes subject to stop codon readthrough revealed that 97% of readthrough codons were perfectly conserved; substitution of an alternative stop codon was rare and involved only UAA and UAG (Jungreis *et al.* 2011). This suggests that the three codons are not functionally identical in the context of readthrough events, but that UAA and UAG may be similar.

Many of the longer carboxy readthrough extensions have in common a distinct pattern of conservation: regions of low complexity, variable conservation, and variable length interspersed with regions of protein sequence conservation. They show characteristics of intrinsically disordered protein regions, which confer structural flexibility and may be characteristic of many members of protein complexes (reviewed in Mészáros *et al.* 2011). For these cases, the carboxy extension due to stop-codon translational readthrough may result in an increased repertoire of protein-protein interactions. An interesting example is *caps*, which encodes a leucine-rich transmembrane protein and is similar to *trn*. The 240-aa extension of the Caps protein expands the similarity of the two proteins into a region with this pattern of conservation that is present in the unextended region of the Trn protein (Figure S2).

Jungreis *et al.* (2011) also used the PhyloCSF algorithm to identify stop-codon readthrough candidates in mammals, nematodes, fungi, and other insects. Although examples of predicted nonselenocysteine readthroughs were found in other phylogenetic groups, only in insects were they found to be common. Use of the three stop codons was not uniform: in many species, only UGA readthroughs were observed and UAA readthroughs were found only in Dipterans. Using a Z-curve reading-frame bias analysis that provided an estimation of the total frequency of readthroughs, Jungreis *et al.* (2011) assessed 25 species in a broad phylogenetic range. They concluded that within the animal kingdom, abundant readthough may be confined to insects and crustacea.

#### FlyBase and GenBank flags:

Genes with transcripts subject to stop-codon readthrough are identified by a specific SO term (Table 1). At the transcript level, comments are included in FlyBase transcript reports and double readthroughs are identified (Table S1). A translation exception flag appears in the GenBank RefSeq transcript and protein entries (Table S2). In the sequences of the predicted proteins, an “X” appears at the position(s) of the suppressed stop codon(s). The proposed bulk data flag is of the “translation exception” type (Table 2).

### Exceptional translation (4): a single translational frameshift is highly conserved

*D. melanogaster* has a single example of translational frameshifting, involving the gene *Oda* (Ornithine decarboxylase antizyme) (Ivanov *et al.* 1998). This event is part of a mechanism of polyamine autoregulation that is conserved from yeast through mammals (Ivanov *et al.* 2000; Olsen and Zetter 2011). The ornithine decarboxylase antizyme regulates the activity of the enzyme ornithine decarboxylase (ODC), a key enzyme in the synthesis of polyamines. The *Oda* transcript does not code for a functional antizyme without an additional regulatory step: the reading frame changes in the second exon. High polyamine concentrations drive a +1 ribosomal frameshift, thus the production of functional Oda protein is sensitive to polyamine levels.

#### FlyBase and GenBank flags:

The *Oda* gene report includes an appropriate SO term (Table 1), and reports for the transcripts include an explanatory comment (Table S1). A “ribosomal_slippage” flag appears in the GenBank RefSeq transcript and protein entries (Table S2). The proposed bulk data flag is of the “translation exception” type (Table 2).

### Exceptional translation (5): mitochondria play by their own rules

There are 13 protein-coding genes mapped to the *D. melanogaster* mitochondrial genome (mitochondrial genes are flagged by the SO term “mt_gene,” SO:0000088). Because mitochondria use an alternative genetic code, all mitochondrial protein-coding transcripts are flagged as translation exceptions (see http://www.ncbi.nlm.nih.gov/Taxonomy/Utils/wprintgc.cgi#SG5 for nonstandard codon usage specific to invertebrate mitochondria). In addition, some mitochondrial genes have an incomplete stop codon. This is a stop codon that is not entirely encoded in the genome: it is completed upon post-transcriptional 3′ polyadenylation. The transcripts of three mitochondrial genes (*mt:CoII*, *mt:ND5*, *mt:ND4*) have this type of structure and have an additional translation exception comment referring to the stop codon exception. For one mitochondrial gene (*mt:CoI*) the start codon has not been identified. Mitochondrial translation is known to use noncanonical start codons, but the sequence preceding *mt:CoI* does not contain any of the identified alternative codons in the correct ORF. The transcript for this gene is flagged with the comment “start codon not determined."

### Conclusion

The annotation of the *D. melanogaster* genome has been greatly facilitated by access to both extensive sets of high throughput data and a large body of gene-specific research. Combined with expert manual assessment of each gene model, this has allowed FlyBase to compile a uniquely detailed and nuanced gene model annotation set, which continues to improve. Unfortunately, some of the most interesting aspects of our knowledge of the *D. melanogaster* genome are difficult to leverage to inform the annotations of other species.

The subjects of this article, the rule-benders or exceptional cases, largely identify biological phenomena that create problems for automated gene prediction algorithms. Although some cases among the rule-benders would present more difficulties than others, it may be feasible to incorporate automated second-pass steps to identify many exceptional cases. A number of highly conserved phenomena are well defined; they affect relatively few genes, but these may be straightforward to identify.

A parallel approach would be a gene model pipeline that is largely automated, but that allows incorporation of manual corrections and additions. Ideally, this would be an ongoing process as more is learned about the variability and flexibility of the genome. If an efficient system for expert review of submissions were developed, then input from a variety of sources, such as researchers interested in a group of genes across many species, undergraduate annotation projects, and even specialist crowd-sourcing, could be encouraged.

## Supplementary Material

Supporting Information
