# Supplementary material for: Gene Model Annotations for Drosophila melanogaster: The Rule-Benders
Source: G3 (Bethesda). 2015 Jun 24;5(8):1737–49. doi: 10.1534/g3.115.018937 (PMC4528330; doi:10.1534/g3.115.018937)
Supplement: Supporting Information [file supp_5_8_1737__index.html]

Gene Model Annotations for Drosophila melanogaster: The Rule-Benders — Supporting Information 

# Gene Model Annotations for *Drosophila melanogaster*: The Rule-Benders

## Supporting Information for Crosby *et al.*, 2015

**Files in this Data Supplement:**

- Supporting Information - Figures S1-S2, Tables S1-S3, and Files S1-S5 (PDF, 446 KB)
- Figure S1 - Polycistronic locus with monocistronic, dicistronic and tricistronic alternative transcripts. (PDF, 152 KB)
- Figure S2 - Conservation of protein sequence beyond stop-codon readthroughs. (PDF, 292 KB)
- Table S1 - Standardized comments used by FlyBase for flagging exceptional transcripts. (PDF, 137 KB)
- Table S2 - GenBank flags used in transcript and protein RefSeq entries. (PDF, 132 KB)
- Table S3 - Genes annotated with a non-AUG translation start in release 6.04. (PDF, 139 KB)
- File S1 - Complete listing of polycistronic loci. (.xlsx, 34 KB)
- File S2 - Complete listing of genes that share exons with other genes. (.xlsx, 17 KB)
- File S3 - Complete listing of genes with multiphasic exons. (.xlsx, 29 KB)
- File S4 - Listing of all annotated introns with non-canonical splices. (.xlsx, 15 KB)
- File S5 - Complete listing of genes annotated with a stop-codon readthrough. (.xlsx, 27 KB)
