# Supplementary material for: Gene Model Annotations for Drosophila melanogaster: The Rule-Benders
Source: G3 (Bethesda). 2015 Jun 24;5(8):1737–49. doi: 10.1534/g3.115.018937 (PMC4528330; doi:10.1534/g3.115.018937)
Supplement: Supporting Information [file supp_g3.115.018937_018937SI.pdf]

G3 · Genes | Genomes | Genetics

SUPPORTING INFORMATION

Gene Model Annotations for *Drosophila melanogaster*: The Rule-Benders

Madeline A. Crosby*[1], L. Sian Gramates*, Gilberto dos Santos*, Beverley B. Matthews*, Susan E. St. Pierre*, Pinglei Zhou*, Andrew J. Schroeder*, Kathleen Falls*, David B. Emmert*, Susan M. Russo*, William M. Gelbart*, and the FlyBase Consortium

*Department of Molecular and Cellular Biology, Harvard University, Cambridge, MA 02138

[1]Corresponding author: FlyBase, Biological Laboratories. Harvard University, 16 Divinity Avenue, Cambridge, MA 02138, 617.495.9925, crosby@morgan.harvard.edu

DOI: 10.1534/g3.115.018937

Figures and tables in Supporting Information (this file)

Figure S1. Polycistronic locus with monocistronic, dicistronic and tricistronic alternative transcripts.

Figure S2. Conservation of protein sequence beyond stop-codon readthroughs.

Table S1: Standardized comments used by FlyBase for flagging exceptional transcripts

Table S2: GenBank flags used in transcript and protein RefSeq entries

Table S3: Genes annotated with a non-AUG translation start in release 6.04

Supporting Information submitted as separate files:

File S1 Complete listing of polycistronic loci.
File S2 Complete listing of genes that share exons with other genes.
File S3 Complete listing of genes with multiphasic exons.
File S4 Listing of all annotated introns with non-canonical splices.
File S5 Complete listing of genes annotated with a stop-codon readthrough.

Available for download as Excel files at www.g3journal.org/lookup/suppl/doi:10.1534/g3.115.018937/-/DC1

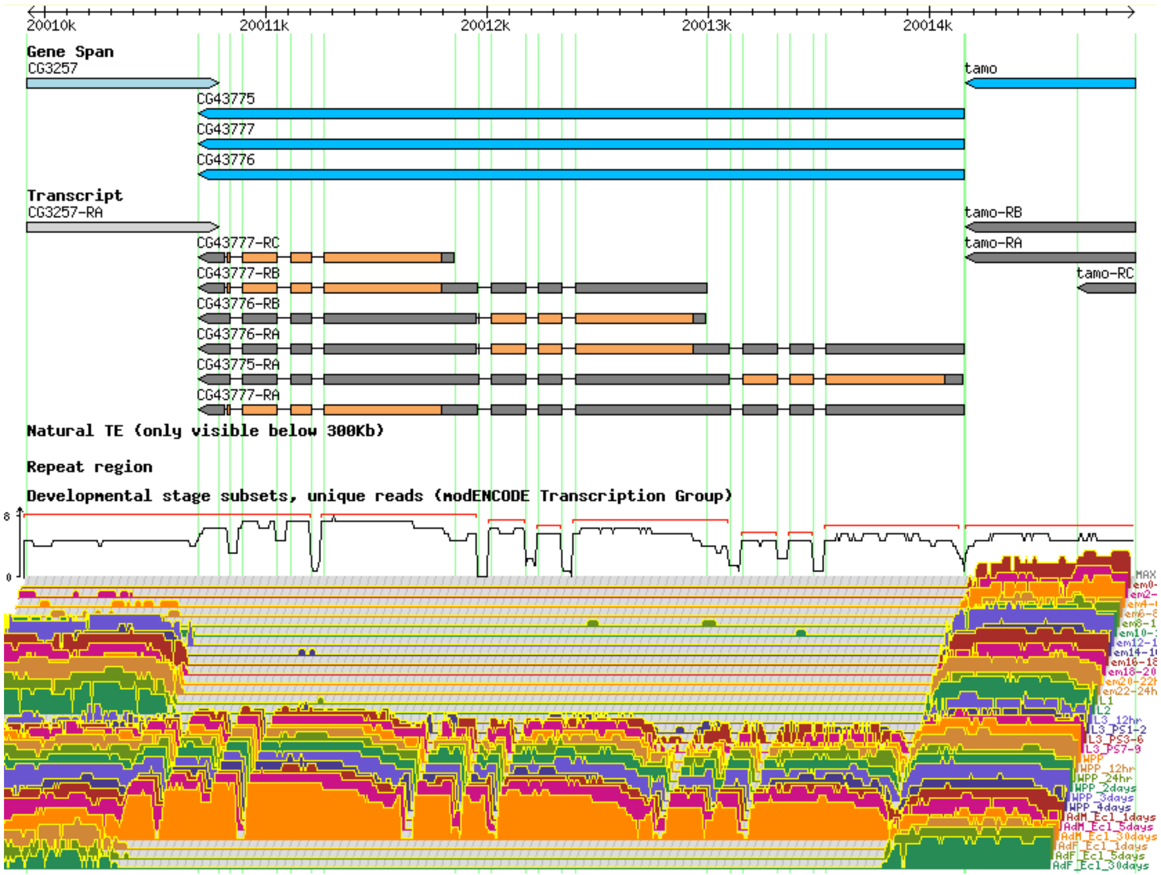

Figure S1 Polycistronic locus with monocistronic, dicistronic and tricistronic alternative transcripts. All annotated introns are supported by cDNA and RNA-Seq junction data; downstream transcription start sites are supported by RAMPAGE TSS data. A GBrowse view showing (top to bottom) the gene extents and the gene models; unstranded RNA-Seq coverage data corresponding to a developmental series (early embryos, top, to adults, bottom). More information on data presented in GBrowse may be found at http://flybase.org/wiki/FlyBase:GBrowse_Tracks#General.

```
CLUSTAL 2.1 multiple sequence alignment


Dmel_trn-PB       IRETIKGGLWGNSALGRKEREYQKTFCDEDYMSRHQHHPCSLGIHSTFPNTYTAPHH--P 58
Dpse_trn          IREYLKGGLWGSSALGRKEREYQKTFCDEDYMARHQHHPCSLGIHSTFPNTYTAPHH--P 58
Dvir_trn          IREYLKGGVWGNSALGRKEREYQKTFCDEDYMSRLQHHPCSLGIHSTFPNTYTAPHQATA 60
Dgri_trn          IREYLKGGLWGNSALGRKEREYQKTFCDEDYMSRLQHHPCSLGIHSTFPNTYTAPHQTTA 60
Dmel_caps-PD      IREMLKG----HSALGRKEREYQKTFSDEEYMSRPP-PGGGG-VHPAAGG--------YP 46
Dpse_caps         IREMLKG----HSALGRKEREYQKTFSDEEYMTRPP-PGCGG-VHPAAA---------YP 45
Dgri_caps         IGELFKG----HSALGRKEREYQKTFSDEDYMTRPPAPGCVGGVHPGSG---------YP 47
Dvir_caps         IREMLKG----HSALGRKEREYQKTFSDEEYMTRPP-PGCGG-VHPASG---------YP 45
                  * * :**     **************.**:**:*         :*.              .

Dmel_trn-PB       GAT----HHYGMCP-MPVNDLGAI--DPQQKFQQLVVP-----------TATMIS-EKK 98
Dpse_trn          SAPG---HHYGMCTGMPINDLNAAGGDPQQKFQQLQVP-----------TGSLMN-EKK 102
Dvir_trn          SAP---HHHYGMCP-MPINDLNAV--DGQHKFQQLQVP----------ITATLMH-EKK 102
Dgri_trn          AAAAAAHHHYGMCP-MPINDLNSG--DGQHKLQQLQVPA---------MSATLLHTEQK 107
Dmel_caps-PD      YIAG-------NSRMIPVTELXLEAPPPPQLRGRGG-----------GGGASTAS--GA 85
Dpse_caps         CSQSQ----YMGSRPIPVTELXLEAPPPPQMRGRGGIASTTTTTTTSSGSGSSSGSTAAP 101
Dgri_caps         CPSSYNTTQYLGSRPIPVTELXLEVPPPPQLRGRGG------APTSH---------GAS 91
Dvir_caps         CPSGINSSQYLGSRSIPVTELXLEAPPPPQLRGRGG------APSSHNGPMASTTSGTP 98
                              .  :*:.:*        :   :

Dmel_trn-PB       LNNNKALVSQGAIDDSASFVLHMKSATMGRDVHQ-----QNPQ---------------- 136
Dpse_trn          LNNNKALASQAAIDDSASFVLHMKSATLARDHLQ-----QHPHPHPQQHPQQH--QHQSG 155
Dvir_trn          LNNNKSLA--GSVDDSASFVLHMKTATMGREQQQ-----AQLQQQHQLQQQQL--QQQHP 153
Dgri_trn          LNNNKALA--GSIDDSANFVLHMKTATMNRDHQQ-----QQQQQQLQQQQQQLPHQQQHP 160
Dmel_caps-PD      VQQLQVPSAVDQAS--NSFAQLSHIHYMTNNGQQ---------QQAQQQQSTSKMHHSQ 133
Dpse_caps         LQQLQVPSPVDHAAAASSFAQLSHIHYMTNN------------QTIASPMTNPQMHHSQ 148
Dgri_caps         LQQLQIVPSAIDA--HAPFAQLSHIHYMTNNPLTTASSAASSAAAASTTATTTPRSHHSQ 149
Dvir_caps         LQQLQ-VPSAVDAS-HAPFAQLSHIHYMTNNPQS---------AATTTATTTPRSHHSQ 146
                  ::: :             *.   :   :  .:

Dmel_trn-PB       ----LNHYTKPQFLSATATVGDSCYS----YADVPMVHGAPLGGP--NQPQLRLTQEHFK 186
Dpse_trn          VQSKLNHYTKPQFLSATAAVGDSCYS----YADVPMVHAAPLGAAPQLQPQLRLTHEHFK 211
Dvir_trn          HQSKLNHYTKPQFLAATATVADSCYS----YADVPMVHAP---------QQLRVTHEHFK 200
Dgri_trn          QQSKLNHYTKPQFLAATATVADSCYS----YADVPLVHAP---------QQLRITHEHFK 207
Dmel_caps-PD      QDMRLLACNGGKPLNA-TSLPRHRP---PVVQESTLSHYSQP-----LANGIRLTQDHFN 184
Dpse_caps         QDMRLLANGGGKTLNA-ASLPRHR------MQESTLSHYSQPLA---LANGIRLTQDHFN 198
Dgri_caps         QDMRLLANG--KALGLNASLPRHMAGRQCGVQESTLSHYSQPVG-------IRLTQDHFN 200
Dvir_caps         QDMRLLANGGGKVLGLNASLPRHMAGRQCGVQESTLSHYSQPLAN--GHAGIRLTQDHFN 204
                      *      : *   :::            : .: * .           :*:*::**:

Dmel_trn-PB       QR-----------------------------ELYDQEMGS-EILDHNYIYSNTHYSMPLE 216
Dpse_trn          HRVAGT-----------------------GEHYDNEVNS-EILDPNYIYSNAHYSMPLE 246
Dvir_trn          QREQRE-----------------------PRDFEADAINGEMMDPNYIYSNAHYSLPLE 236
Dgri_trn          QRE----------------------------RDFDNENPLGEMMDPNYIYSNAHYSLPLE 239
Dmel_caps-PD      HNQNQ--------------------SHNQHYGG-VYAKPCDAMSEPGYIHNNSHYSLPLD 223
Dpse_caps         HNHSHGHGHGHGHGHGHPHSLGHPHSHTTHNGGGVYAKACDAMTEPGYIHNNSHYSLPLD 258
Dgri_caps         HN-----------------------------GGVYAKPCDAMTDPGYIHNNSHYSLPLD 230
Dvir_caps         HNGIGG------------------------GAGVYAKPCDAMAEAGYIHNNSHYSLPLD 239
                  :.                                        : : .**:.*:***:**:

Dmel_trn-PB       QLGRSKTPTPPPMPPALPLRNGL------CATTGRRSFQQKSASQKQQQNNNTLRQFTHX 270
Dpse_trn          QMGRSKTPTPPPMPPALPLRNGL------CATTGRRSFQQKSA------NNNTLRQFTHX 294
Dvir_trn          QMGRNKTPTPPPVPPALPLRNGL------CATTGRRSFQQK-TPAHNN-NTSTMRQFTHX 288
Dgri_trn          QMGRSKTPTPPPLPPALPLRNGL------CATTGRRSLQHRPTPATN-----TLRQFTHX 288
Dmel_caps-PD      HDLPP-SPTPTPPPPALPLRNGVGMALIHGNTTGRRSFN-------NNNNVSTLSNNNHH 275
Dpse_caps         HDLPP-SPTPTPPPPALPLRNGVGMALIHGNTTGRRSFNSNSNS--NNNNVATLSNNNHH 315
Dgri_caps         HDMPP-SPTPTPPPPALPLRNGLGMALVHGNTTGRRSFNSSSSNSNNNNNVATLSNNNHN 289
Dvir_caps         HDMPP-SPTPTPPPPALPLRNGMGMALVHGNTTGRRSFNS------NNNNVATLSNNNHN 292
                  :     :***.* *********:        ******::             *: : .*

Dmel_trn-PB       -----------------SSTYRRRQLSIYA--- 283
Dpse_trn          ----------------STVDYRRRQLSIYA--- 308
Dvir_trn          ----------------SSEHYRRRQLSIYA--- 302
Dgri_trn          ----------------SSEHYRRRQLSIFA--- 302
Dmel_caps-PD      ----GIGGGVG-VAVGGTVGNNNGSLRRYH--- 300
Dpse_caps         ----GGGGGGGGGAILASSNNNGSLRRYH--- 341
Dgri_caps         ----GLVLANG---CSG-INNNNGSLRRYH--- 311
Dvir_caps         GVGGGAVLVNGNYNTNGSNNNNNGSLRRYH--- 322
                                       .. .*  :
```

Figure S2 Conservation of protein sequence beyond stop-codon readthroughs. Clustal alignment (http://www.ebi.ac.uk/Tools/msa/clustalw2/) of the carboxy end of *tartan* gene proteins from several Drosophila species (if annotated with a short read-though extension) and the predicted carboxy end of *caps* gene proteins (if annotated with a long read-through extension) from several Drosophila species. The *caps* readthrough (denoted by 'X') is at alignment position 82; the *trn* readthrough is at alignment position 300. Note regions of low complexity, variable conservation and variable length interspersed with regions of protein sequence conservation.

Table S1 Standardized comments used by FlyBase for flagging exceptional transcripts

**FlyBase transcript-associated standardized comments**
Polycistronic transcript.
Dicistronic transcript.
Dicistronic transcript; alternative monocistronic transcript(s) exist.
Monocistronic transcript; alternative dicistronic transcript(s) exist.
Unconventional splice site postulated ([*splice donor-acceptor specified]*).
Unconventional splice site invoked ([*splice donor-acceptor specified]*): sequence altered due to transposon insertion; this splice may not occur in vivo.
Unconventional splice site(s) invoked due to gap in genomic sequence; this splice does not occur in vivo.
Unconventional splice site(s) invoked ([*splice donor-acceptor])*; within a dynamic region of nested TEs that may differ in different strains; this splice may not occur in vivo.
Unconventional splice site postulated: transcript subject to HAC1-type intron splice site recognition and cleavage.
Trans-spliced.
Unconventional translation start postulated ([*codon specified]* encoding Met).
Stop-codon suppression (UGA as Sec) postulated; reflected in aa sequence of predicted polypeptide.
Stop-codon suppression postulated *([codon specified])*; reflected in aa sequence of predicted polypeptide.
Double stop-codon suppression postulated *([codons specified])*; reflected in aa sequence of predicted polypeptide.
Translational frameshifting postulated: +1 frameshift reflected in aa sequence of predicted polypeptide.
TAA stop codon is completed by the addition of 3' A residues to the mRNA.
Start codon not determined.

Table S2   GenBank flags used in transcript and protein RefSeq entries

**GenBank flags for exceptional cases**
gene /exception="dicistronic gene" [transcript RefSeq entries only]
CDS /exception="nonconsensus splice site"
CDS /trans_splicing
CDS /note="non-AUG ([*codon specified*]) translation initiation"
CDS /transl_except=(pos:*x*..*y*,aa:Met)
CDS /transl_except=(pos:*x*..*y*,aa:Sec)
CDS /transl_except=(pos:*x*..*y*,aa:OTHER)
CDS /ribosomal_slippage
CDS /transl_table=5
CDS /transl_except=(pos:*x*,aa:TERM)
CDS /note="TAA stop codon is completed by the addition of 3' A"
CDS /note="start codon not determined"

Table S3 Genes annotated with a non-AUG translation start in release 6.04

Gene	Start codon	Reference (FlyBase Reference ID)
Eip74EF	CUG	Burtis *et al* 1990 (FBrf0051390), Boyd and Thummel 1993 (FBrf0064374)
cpo	CUG	Bellen *et al* 1992 (FBrf0056119)
ewg	CUG	de Simone and White 1993 (FBrf0059052)
Eip78C	CUG	Stone and Thummel 1993 (FBrf0064719)
Syn	CUG	Klagges *et al* 1996 (FBrf0087510)
att-ORFA	CUG	Madigan *et al* 1996 (FBrf0089733)
Fmr1	CUG	Beerman and Jongens 2011 (FBrf0213401)
Trpγ	CUG	FlyBase analysis
CG4629	CUG	FlyBase analysis
CG11076	CUG	FlyBase analysis
CG16890	CUG	FlyBase analysis
Cha	GUG	Sugihara *et* al. 1990 (FBrf0052176)
Akt1	GUG	FlyBase analysis
Klp54D	ACG	Andjelkovic *et* al. 1995 (FBrf0079853)
NAT1	AUU	Takahashi *et* al 2005 (FBrf0184018)
Gsc	AUU	FlyBase analysis
CG11836	AUU	FlyBase analysis
Wnk	AUU	FlyBase analysis
Sh	AUU	FlyBase analysis
CG14989	UUG	FlyBase analysis
Jwa	UUG	FlyBase analysis
sol	UUG	FlyBase analysis
CG43778	UUG	FlyBase analysis
CG2162	AUC	FlyBase analysis
CG43921	AUC	FlyBase analysis
CG30334	AUC	FlyBase analysis
CG43273	AUC	FlyBase analysis
