# Supplementary material for: Gene Model Annotations for Drosophila melanogaster: The Rule-Benders
Source: G3 (Bethesda). 2015 Jun 24;5(8):1737–49. doi: 10.1534/g3.115.018937 (PMC4528330; doi:10.1534/g3.115.018937)
Supplement: Supporting Information [file supp_g3.115.018937_FigureS1.pdf]

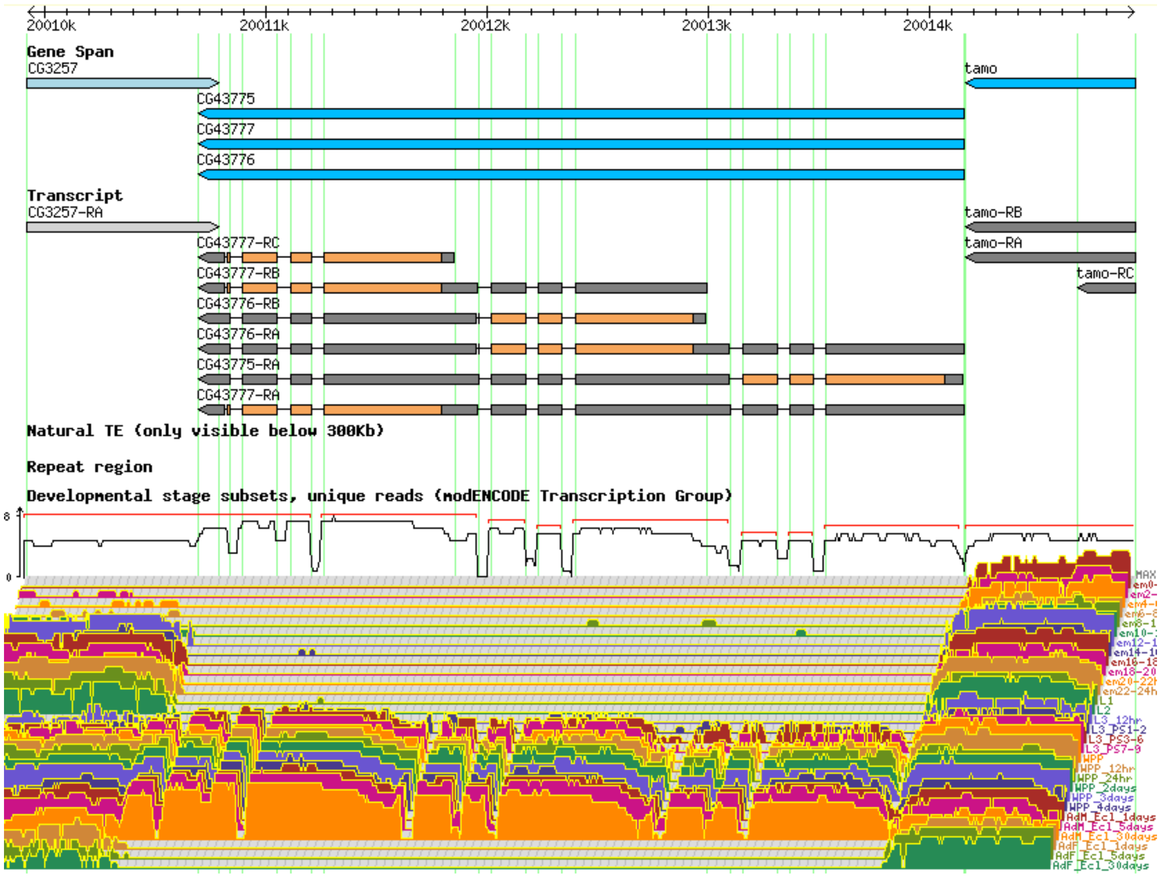

Figure S1 Polycistronic locus with monocistronic, dicistronic and tricistronic alternative transcripts. All annotated introns are supported by cDNA and RNA-Seq junction data; downstream transcription start sites are supported by RAMPAGE TSS data. A GBrowse view showing (top to bottom) the gene extents and the gene models; unstranded RNA-Seq coverage data corresponding to a developmental series (early embryos, top, to adults, bottom). More information on data presented in GBrowse may be found at http://flybase.org/wiki/FlyBase:GBrowse_Tracks#General.
