# Supplementary material for: Gene Model Annotations for Drosophila melanogaster: The Rule-Benders
Source: G3 (Bethesda). 2015 Jun 24;5(8):1737–49. doi: 10.1534/g3.115.018937 (PMC4528330; doi:10.1534/g3.115.018937)
Supplement: Supporting Information [file supp_g3.115.018937_FigureS2.pdf]

```
CLUSTAL 2.1 multiple sequence alignment


Dmel_trn-PB      IRETIKGGLWGNSALGRKEREYQKTFCDEDYMSRHQHHPCSLGIHSTFPNTYTAPHH--P 58
Dpse_trn         IREYLKGGLWGSSALGRKEREYQKTFCDEDYMARHQHHPCSLGIHSTFPNTYTAPHH--P 58
Dvir_trn         IREYLKGGVWGNSALGRKEREYQKTFCDEDYMSRLQHHPCSLGIHSTFPNTYTAPHQATA 60
Dgri_trn         IREYLKGGLWGNSALGRKEREYQKTFCDEDYMSRLQHHPCSLGIHSTFPNTYTAPHQTTA 60
Dmel_caps-PD     IREMLKG----HSALGRKEREYQKTFSDEEYMSRPP-PGGGG-VHPAAGG--------YP 46
Dpse_caps        IREMLKG----HSALGRKEREYQKTFSDEEYMTRPP-PGCGG-VHPAAA---------YP 45
Dgri_caps        IGELFKG----HSALGRKEREYQKTFSDEDYMTRPPAPGCVGGVHPGSG---------YP 47
Dvir_caps        IREMLKG----HSALGRKEREYQKTFSDEEYMTRPP-PGCGG-VHPASG---------YP 45
                 * * :**     **************.**:**:*         :*.             .

Dmel_trn-PB      GAT----HHYGMCP-MPVNDLGAI--DPQQKFQQLVVP------------TATMIS-EKK 98
Dpse_trn         SAPG---HHYGMCTGMPINDLNAAGGDPQQKFQQLQVP------------TGSLMN-EKK 102
Dvir_trn         SAP---HHHYGMCP-MPINDLNAV--DGQHKFQQLQVP-----------ITATLMH-EKK 102
Dgri_trn         AAAAAAHHHYGMCP-MPINDLNSG--DGQHKLQQLQVPA----------MSATLLHTEQK 107
Dmel_caps-PD     YIAG-------NSRMIPVTELXLEAPPPPQLRGRGG------------GGGASTAS--GA 85
Dpse_caps        CSQSQ----YMGSRPIPVTELXLEAPPPPQMRGRGGIASTTTTTTTSSGSGSSSGSTAAP 101
Dgri_caps        CPSSYNTTQYLGSRPIPVTELXLEVPPPPQLRGRGG-------APTSH---------GAS 91
Dvir_caps        CPSGINSSQYLGSRSIPVTELXLEAPPPPQLRGRGG-------APSSHNGPMASTTSGTP 98
                             .  :*:.:*        :   :

Dmel_trn-PB      LNNNKALVSQGAIDDSASFVLHMKSATMGRDVHQ-----QNPQ----------------- 136
Dpse_trn         LNNNKALASQAAIDDSASFVLHMKSATLARDHLQ-----QHPHPHPQQHPQQH--QHQSG 155
Dvir_trn         LNNNKSLA--GSVDDSASFVLHMKTATMGREQQQ-----AQLQQQHQLQQQQL--QQQHP 153
Dgri_trn         LNNNKALA--GSIDDSANFVLHMKTATMNRDHQQ-----QQQQQQLQQQQQQLPHQQQHP 160
Dmel_caps-PD     VQQLQVPSAVDQAS--NSFAQLSHIHYMTNNGQQ----------QQAQQQQSTSKMHHSQ 133
Dpse_caps        LQQLQVPSPVDHAAAASSFAQLSHIHYMTNN-------------QTIASPMTNPQMHHSQ 148
Dgri_caps        LQQLQIVPSAIDA--HAPFAQLSHIHYMTNNPLTTASSAASSAAAASTTATTTPRSHHSQ 149
Dvir_caps        LQQLQ-VPSAVDAS-HAPFAQLSHIHYMTNNPQS----------AATTTATTTPRSHHSQ 146
                 ::: :             *.   :   : .:

Dmel_trn-PB      ----LNHYTKPQFLSATATVGDSCYS----YADVPMVHGAPLGGP--NQPQLRLTQEHFK 186
Dpse_trn         VQSKLNHYTKPQFLSATAAVGDSCYS----YADVPMVHAAPLGAAPQLQPQLRLTHEHFK 211
Dvir_trn         HQSKLNHYTKPQFLAATATVADSCYS----YADVPMVHAP---------QQLRVTHEHFK 200
Dgri_trn         QQSKLNHYTKPQFLAATATVADSCYS----YADVPLVHAP---------QQLRITHEHFK 207
Dmel_caps-PD     QDMRLLACNGGKPLNA-TSLPRHRP---PVVQESTLSHYSQP-----LANGIRLTQDHFN 184
Dpse_caps        QDMRLLANGGGKTLNA-ASLPRHR------MQESTLSHYSQPLA---LANGIRLTQDHFN 198
Dgri_caps        QDMRLLANG--KALGLNASLPRHMAGRQCGVQESTLSHYSQPVG-------IRLTQDHFN 200
Dvir_caps        QDMRLLANGGGKVLGLNASLPRHMAGRQCGVQESTLSHYSQPLAN--GHAGIRLTQDHFN 204
                     *      : *   :::            : .: * .           :*:*::**:

Dmel_trn-PB      QR-----------------------------ELYDQEMGS-EILDHNYIYSNTHYSMPLE 216
Dpse_trn         HRVAGT------------------------GEHYDNEVNS-EILDPNYIYSNAHYSMPLE 246
Dvir_trn         QREQRE------------------------PRDFEADAINGEMMDPNYIYSNAHYSLPLE 236
Dgri_trn         QRE----------------------------RDFDNENPLGEMMDPNYIYSNAHYSLPLE 239
Dmel_caps-PD     HNQNQ--------------------SHNQHYGG-VYAKPCDAMSEPGYIHNNSHYSLPLD 223
Dpse_caps        HNHSHGHGHGHGHGHGHPHSLGHPHSHTTHNGGGVYAKACDAMTEPGYIHNNSHYSLPLD 258
Dgri_caps        HN------------------------------GGVYAKPCDAMTDPGYIHNNSHYSLPLD 230
Dvir_caps        HNGIGG-------------------------GAGVYAKPCDAMAEAGYIHNNSHYSLPLD 239
                 :.                                        : : .**:.*:***:**:

Dmel_trn-PB      QLGRSKTPTPPPMPPALPLRNGL------CATTGRRSFQQKSASQKQQQNNNTLRQFTHX 270
Dpse_trn         QMGRSKTPTPPPMPPALPLRNGL------CATTGRRSFQQKSA------NNNTLRQFTHX 294
Dvir_trn         QMGRNKTPTPPPVPPALPLRNGL------CATTGRRSFQQK-TPAHNN-NTSTMRQFTHX 288
Dgri_trn         QMGRSKTPTPPPLPPALPLRNGL------CATTGRRSLQHRPTPATN-----TLRQFTHX 288
Dmel_caps-PD     HDLPP-SPTPTPPPPALPLRNGVGMALIHGNTTGRRSFN-------NNNNVSTLSNNNHH 275
Dpse_caps        HDLPP-SPTPTPPPPALPLRNGVGMALIHGNTTGRRSFNSNSNS--NNNNVATLSNNNHH 315
Dgri_caps        HDMPP-SPTPTPPPPALPLRNGLGMALVHGNTTGRRSFNSSSSNSNNNNNVATLSNNNHN 289
Dvir_caps        HDMPP-SPTPTPPPPALPLRNGMGMALVHGNTTGRRSFNS------NNNNVATLSNNNHN 292
                 :     :***.* *********:        ******::             *: : .*

Dmel_trn-PB      -----------------SSTYRRRQLSIYA--- 283
Dpse_trn         ----------------STVDYRRRQLSIYA--- 308
Dvir_trn         ----------------SSEHYRRRQLSIYA--- 302
Dgri_trn         ----------------SSEHYRRRQLSIFA--- 302
Dmel_caps-PD     ----GIGGGVG-VAVGGTVGNNNGSLRRYH--- 300
Dpse_caps        ----GGGGGGGGGGAILASSNNNGSLRRYH--- 341
Dgri_caps        ----GLVLANG---CSG-INNNNGSLRRYH--- 311
Dvir_caps        GVGGGAVLVNGNYNTNGSNNNNNGSLRRYH--- 322
                                      .. .*  :
```

Figure S2 Conservation of protein sequence beyond stop-codon readthroughs. Clustal alignment (http://www.ebi.ac.uk/Tools/msa/clustalw2/) of the carboxy end of *tartan* gene proteins from several Drosophila species (if annotated with a short read-though extension) and the predicted carboxy end of *caps* gene proteins (if annotated with a long read-through extension) from several Drosophila species. The *caps* readthrough (denoted by 'X') is at alignment position 82; the *trn* readthrough is at alignment position 300. Note regions of low complexity, variable conservation and variable length interspersed with regions of protein sequence conservation.
