# Supplementary material for: Gene Model Annotations for Drosophila melanogaster: The Rule-Benders
Source: G3 (Bethesda). 2015 Jun 24;5(8):1737–49. doi: 10.1534/g3.115.018937 (PMC4528330; doi:10.1534/g3.115.018937)
Supplement: Supporting Information [file supp_g3.115.018937_TableS1.pdf]

Table S1 Standardized comments used by FlyBase for flagging exceptional transcripts

**FlyBase transcript-associated standardized comments**
Polycistronic transcript.
Dicistronic transcript.
Dicistronic transcript; alternative monocistronic transcript(s) exist.
Monocistronic transcript; alternative dicistronic transcript(s) exist.
Unconventional splice site postulated ([*splice donor-acceptor specified]*).
Unconventional splice site invoked ([*splice donor-acceptor specified]*): sequence altered due to transposon insertion; this splice may not occur in vivo.
Unconventional splice site(s) invoked due to gap in genomic sequence; this splice does not occur in vivo.
Unconventional splice site(s) invoked ([*splice donor-acceptor])*; within a dynamic region of nested TEs that may differ in different strains; this splice may not occur in vivo.
Unconventional splice site postulated: transcript subject to HAC1-type intron splice site recognition and cleavage.
Trans-spliced.
Unconventional translation start postulated ([*codon specified]* encoding Met).
Stop-codon suppression (UGA as Sec) postulated; reflected in aa sequence of predicted polypeptide.
Stop-codon suppression postulated *([codon specified])*; reflected in aa sequence of predicted polypeptide.
Double stop-codon suppression postulated *([codons specified])*; reflected in aa sequence of predicted polypeptide.
Translational frameshifting postulated: +1 frameshift reflected in aa sequence of predicted polypeptide.
TAA stop codon is completed by the addition of 3' A residues to the mRNA.
Start codon not determined.
