# Supplementary material for: Gene Model Annotations for Drosophila melanogaster: The Rule-Benders
Source: G3 (Bethesda). 2015 Jun 24;5(8):1737–49. doi: 10.1534/g3.115.018937 (PMC4528330; doi:10.1534/g3.115.018937)
Supplement: Supporting Information [file supp_g3.115.018937_TableS2.pdf]

Table S2 GenBank flags used in transcript and protein RefSeq entries

**GenBank flags for exceptional cases**
gene /exception="dicistronic gene" [transcript RefSeq entries only]
CDS /exception="nonconsensus splice site"
CDS /trans_splicing
CDS /note="non-AUG ([*codon specified*]) translation initiation"
CDS /transl_except=(pos:*x*..*y*,aa:Met)
CDS /transl_except=(pos:*x*..*y*,aa:Sec)
CDS /transl_except=(pos:*x*..*y*,aa:OTHER)
CDS /ribosomal_slippage
CDS /transl_table=5
CDS /transl_except=(pos:*x*,aa:TERM)
CDS /note="TAA stop codon is completed by the addition of 3' A"
CDS /note="start codon not determined"
