# Supplementary material for: Gene Model Annotations for Drosophila melanogaster: The Rule-Benders
Source: G3 (Bethesda). 2015 Jun 24;5(8):1737–49. doi: 10.1534/g3.115.018937 (PMC4528330; doi:10.1534/g3.115.018937)
Supplement: Supporting Information [file supp_g3.115.018937_TableS3.pdf]

Table S3   Genes annotated with a non-AUG translation start in release 6.04

Gene	Start codon	Reference (FlyBase Reference ID)
Eip74EF	CUG	Burtis *et al* 1990 (FBrf0051390), Boyd and Thummel 1993 (FBrf0064374)
cpo	CUG	Bellen *et al* 1992 (FBrf0056119)
ewg	CUG	de Simone and White 1993 (FBrf0059052)
Eip78C	CUG	Stone and Thummel 1993 (FBrf0064719)
Syn	CUG	Klagges *et al* 1996 (FBrf0087510)
att-ORFA	CUG	Madigan *et al* 1996 (FBrf0089733)
Fmr1	CUG	Beerman and Jongens 2011 (FBrf0213401)
Trpγ	CUG	FlyBase analysis
CG4629	CUG	FlyBase analysis
CG11076	CUG	FlyBase analysis
CG16890	CUG	FlyBase analysis
Cha	GUG	Sugihara *et* al. 1990 (FBrf0052176)
Akt1	GUG	FlyBase analysis
Klp54D	ACG	Andjelkovic *et* al. 1995 (FBrf0079853)
NAT1	AUU	Takahashi *et* al 2005 (FBrf0184018)
Gsc	AUU	FlyBase analysis
CG11836	AUU	FlyBase analysis
Wnk	AUU	FlyBase analysis
Sh	AUU	FlyBase analysis
CG14989	UUG	FlyBase analysis
Jwa	UUG	FlyBase analysis
sol	UUG	FlyBase analysis
CG43778	UUG	FlyBase analysis
CG2162	AUC	FlyBase analysis
CG43921	AUC	FlyBase analysis
CG30334	AUC	FlyBase analysis
CG43273	AUC	FlyBase analysis
